# Kynurenine Pathway Metabolites as Mediators of Exercise-Induced Mood Enhancement, Fatigue Resistance, and Neuroprotection

**DOI:** 10.3390/ijms27010129

**Published:** 2025-12-22

**Authors:** Amelia Tero-Vescan, Ruxandra Ștefănescu, Amalia Pușcaș, Mădălina Buț, Bianca-Eugenia Ősz, Mark Slevin

**Affiliations:** 1 Biochemistry Department, Faculty of Medicine in English, George Emil Palade University of Medicine, Pharmacy, Science, and Technology of Târgu Mureș, 540142 Târgu Mureș, Romania; amelia.tero-vescan@umfst.ro (A.T.-V.); amalia.puscas@umfst.ro (A.P.); madalina.but@umfst.ro (M.B.); 2 Pharmacognosy and Phytotherapy Department, Faculty of Pharmacy, George Emil Palade University of Medicine, Pharmacy, Science, and Technology of Târgu Mureș, 540142 Târgu Mureș, Romania; 3 Pharmacology and Clinical Pharmacy Department, Faculty of Pharmacy, George Emil Palade University of Medicine, Pharmacy, Science, and Technology of Târgu Mureș, 540142 Târgu Mureș, Romania; bianca.osz@umfst.ro; 4 Center for Advanced Medical and Pharmaceutical Research, George Emil Palade University of Medicine, Pharmacy, Science, and Technology of Târgu Mureș, 540142 Târgu Mureș, Romania; mark.slevin@umfst.ro

**Keywords:** kynurenine pathway, quinolinic acid, kynurenic acid, irisin, adiponectin, leptin, major depressive disorder, exercise mimetics, HIIT, exercise

## Abstract

Major depressive disorder is increasingly recognized as a metabolic–immune disorder in which chronic inflammation diverts tryptophan (Trp) metabolism toward the kynurenine pathway (KP), reducing serotonin synthesis and producing neurotoxic metabolites such as quinolinic acid (QA). Elevated kynurenine (KYN)/Trp ratios and an altered QA/kynurenic acid (KYNA) balance have been consistently reported in depressed individuals, implicating the KP as a key therapeutic target. Exercise provides a unique, translationally relevant intervention: unlike pharmacological agents acting directly on neurotransmission, contracting skeletal muscle acts as a “kynurenine sink” by inducing kynurenine aminotransferases that convert circulating KYN into neuroprotective KYNA, thereby reducing brain KYN uptake and mitigating excitotoxicity. Clinical studies and meta-analyses confirm that aerobic, resistance, and high-intensity training produce antidepressant effects comparable to pharmacotherapy, while also improving cognition, fatigue tolerance, and cardiometabolic function. Beyond KP remodeling, exercise-induced myokines (irisin, IL-6, BDNF, apelin, FGF21) and adipokines (adiponectin, leptin modulators) coordinate systemic anti-inflammatory and neurotrophic adaptations that enhance resilience and brain plasticity. Furthermore, pharmacological “exercise mimetics” and metabolic modulators, such as PPAR agonists, AMPK activators, NAD^+^ boosters, meldonium, trimetazidine, and adiponectin receptor agonists, may be promising adjuncts for patients with low exercise capacity or metabolic comorbidities. This review provides a novel concept, positioning exercise as a systemic antidepressant that breaks the kynurenine lock of depression. Through proper interpretation of skeletal muscle as an endocrine organ of resilience, we integrate molecular, clinical, and translational findings to show how exercise remodels Trp–KYN metabolism and inflammatory signaling and how pharmacological mimetics may extend these benefits. This perspective consolidates scattered mechanistic and clinical data and outlines a forward-looking therapeutic framework that links exercise and lifestyle, metabolism, and drug discovery. We highlight that re-consideration of our understanding of depression, as a whole-body disorder, should provide new opportunities for precision interventions.

## 1. Introduction

Major depressive disorder (MDD) can be envisioned as a biochemical trap. Affecting women disproportionately (14.4% vs. 11.5% prevalence in men), MDD is often resistant to conventional treatments such as psychotherapy or pharmacotherapy, partly owing to the persistent stigma surrounding mental health but also due to concerns about adverse drug effects, ranging from gastrointestinal disturbances (constipation, diarrhea, nausea) to neurological symptoms (dizziness, headache, insomnia, somnolence) and reduced sexual desire, all of which can compromise both treatment acceptance and long-term compliance [1]. As a result, many patients remain caught in cycles of poor adherence and inadequate recovery.

At the molecular level, inflammation appears to strengthen this trap, whereby proinflammatory cytokines such as interferon-γ (IFN-γ), interleukin-6 (IL-6), and tumor necrosis factor-α (TNF-α) activate indoleamine 2,3-dioxygenase (IDO), diverting tryptophan (Trp) away from serotonin (5-HT) synthesis and into the kynurenine pathway (KP) [2,3]. This “metabolic hijack” decreases 5-HT availability while increasing the levels of neurotoxic metabolites such as quinolinic acid (QA). Elevated kynurenine (KYN)/Trp ratios and disrupted QA/kynurenic acid (KYNA) balance have repeatedly been observed in patients with MDD compared with healthy controls, suggesting that many patients remain locked in a kynurenine-driven cycle of inflammation, neurotransmitter imbalance, and neurotoxicity of sufficient complexity that conventional therapy is often unable to alleviate [4].

Exercise offers a potential, nonsynthetic and broader therapeutic solution since, unlike pharmacological interventions that directly target neurotransmitters, physical activity can recruit and utilize skeletal muscle as a detoxifying organ. Recent meta-analyses have confirmed that aerobic and resistance training exert antidepressant effects comparable to those of pharmacological and behavioral therapies, particularly in mild-to-moderate MDD [5,6]. By upregulating kynurenine aminotransferases (KATs), exercise-trained muscle cells convert circulating KYN into the neuroprotective metabolite KYNA, which cannot cross the blood–brain barrier (BBB). This metabolic diversion lowers systemic KYN, relieves central neurotoxic pressure, and correlates with improvements in mood, resilience, and cognition [7]. In effect, contracting muscle acts as a “kynurenine sink,” clearing the bloodstream of a depressogenic metabolite and opening a biochemical escape route for the brain. In addition, exercise-induced messengers such as irisin, brain-derived neurotrophic factor (BDNF), and adiponectin (myokines and adipokines) contribute by reshaping neuroplasticity, energy metabolism, and the inflammatory tone. For example, higher adiponectin levels are associated with a lower severity of depressive symptoms [8], whereas exercise-induced changes in BDNF and adiponectin correlate with treatment response in resistant forms of depression [9].

Together, these signals transform skeletal muscle into an endocrine organ of resilience, offering systemic protection against the inflammatory/metabolic imbalance that mediates depression.

For this review, a narrative literature search was conducted using the PubMed database to identify peer-reviewed original articles published from 2020 onward, focusing on the kynurenine pathway, exercise-induced metabolic adaptations, and their relevance to mood regulation, fatigue resistance, and neuroprotection. Articles were selected by using keywords “kynurenine pathway”, “exercise”, “neuroprotection” and “mood disorders” based on thematic relevance and mechanistic insight.

This review explores the concept of “breaking the kynurenine trap” through exercise and its biochemical mediators. We highlight how physical activity remodels Trp metabolism, modulates inflammatory signaling, and engages myokine and adipokine networks to influence mood regulation and neuroplasticity. In addition to its antidepressant effects, exercise transforms skeletal muscle into a detoxifying organ that facilitates systemic metabolic recapitulation from neurotoxic imbalance. Importantly, pharmacological exercise mimetics and metabolic modulators may serve as adjunctive tools for patients unable to achieve sufficient physical activity. Such strategies include peroxisome proliferator-activated receptor (PPAR) agonists, AMP-activated protein kinase (AMPK) activators, and nicotinamide adenine dinucleotide (NAD^+^) boosters, as well as clinically available metabolic agents such as meldonium and trimetazidine, which have shown potential to amplify exercise-like adaptations, particularly in individuals with comorbid cardiometabolic disorders or physical limitations [10,11]. By a novel interpretation of muscle as an endocrine organ of resilience and positioning exercise and its mimetics as biochemical keys, we propose that targeting the KP offers a novel, systemic approach to treating MDD, with implications for mood regulation, fatigue resistance, and neuroprotection.

## 2. Trp Metabolism at the Crossroads of Immunity, Neuroinflammation, and Mood Regulation

**Figure 1:**
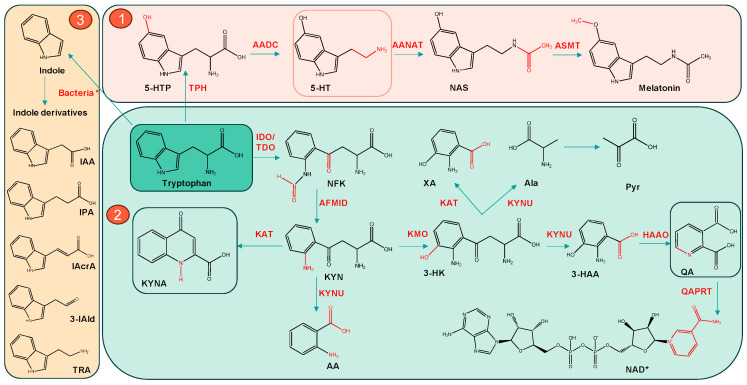
Schematic representation of the major Trp metabolic pathways: ① the 5-HT/melatonin pathway (pink), ② the KP (green), and ③ the indole pathway (yellow, bacterial metabolism). Enzymes are written in red over the arrows and the name of the compounds is black. Trp metabolism enzymes: IDO/TDO—Indoleamine 2,3-dioxygenase/Tryptophan 2,3-dioxygenase, AFMID—Arylformamidase, KAT—Kynurenine aminotransferase, KMO—Kynurenine 3-monooxygenase, KYNU—Kynureninase, HAAO—3-hydroxyanthranilate 3,4-dioxygenase, QAPRT—Quinolinic acid phosphoribosyltransferase, TPH—Tryptophan hydroxylase, AADC—Aromatic L-amino acid decarboxylase, AANAT—Arylalkylamine N-acetyltransferase, ASMT—Acetylserotonin O-methyltransferase, Metabolites: NFK—N-formylkynurenine, KYN—Kynurenine, 3-HK—3-hydroxykynurenine, 3-HAA—3-hydroxyanthranilic acid, KYNA—Kynurenic acid, XA—Xanthurenic acid, AA—Anthranilic acid, QA—Quinolinic acid, NAD^+^—Nicotinamide adenine dinucleotide, Pyr—Pyruvate, Ala—Alanine, 5-HTP—5-hydroxytryptophan, 5-HT—5-hydroxytryptamine (serotonin), NAS—N-acetylserotonin, IAA—Indole-3-acetic acid, IPA—Indole-3-propionic acid, IAcrA—Indole-3-acrylic acid, 3-IAld—Indole-3-aldehyde, TRA—Tryptamine). * *Clostridium sporogenes*, *Lactobacillus reuteri* or *Escherichia coli*.

Trp is an essential amino acid that can be metabolized through the 5-HT pathway and the indole pathway; however, the majority (>95%) of Trp is degraded via the KP, leading to the formation of neuroactive metabolites that influence brain function, ranging from neuroprotective to neurotoxic effects, as well as contributing to inflammation and oxidative stress [12,13]. The overall image of Trp metabolism is shown in Figure 1. The conversion of Trp to 5-HT first requires its hydroxylation to 5-hydroxytryptophan (5-HTP) by tryptophan hydroxylase (TPH), which is then decarboxylated by aromatic L-amino acid decarboxylase (AADC) to form 5-HT. Several metabolites can be generated from 5-HT, including 5-hydroxyindoleacetic acid (5-HIAA) via monoamine oxidase (MAO), N-acetylserotonin (NAS) via arylalkylamine N-acetyltransferase (AANAT), and ultimately melatonin via N-acetylserotonin O-methyltransferase (ASMT) [14].

The key enzymes involved in the KP of Trp metabolism are IDO1, tryptophan 2,3-dioxygenase (TDO), and kynurenine monooxygenase (KMO) [15]. Initially, Trp is converted into N-formylkynurenine (NFK) and subsequently into KYN by arylformamidase (AFMID). KYN can then be metabolized into 3-hydroxykynurenine (3-HK) and anthranilic acid (AA) via KMO and kynureninase (KYNU), respectively. KAT catalyzes the conversion of KYN into KYNA and 3-HK into xanthurenic acid (XA). In parallel, 3-hydroxyanthranilic acid (3-HAA), produced from 3-HK by KYNU, is further metabolized into QA, which serves as a precursor for NAD^+^ biosynthesis via Quinolinic acid phosphoribosyltransferase (QAPRT) [16].

Importantly, the activity of these enzymes, particularly IDO1, is profoundly influenced by the immune system. In MDD, chronic low-grade inflammation and sustained psychosocial stress lead to persistent activation of microglia and, concomitantly, the activation of T helper (TH1) lymphocytes, which secrete proinflammatory cytokines such as IFN-γ, TNF-α, and interleukin-2 (IL-2). These cytokines act as potent inducers of IDO1, thereby shifting Trp metabolism away from 5-HT synthesis toward the KP. The resulting imbalance between neurotoxic (3-HK, QA) and neuroprotective (KYNA) metabolites alters glutamatergic signaling and contributes to neuroinflammation and excitotoxicity, which is a key feature implicated in the pathophysiology of depression, as reviewed in [17]. On the other hand, cytokines associated with the T helper type 2 lymphocyte (TH2) profile, such as interleukin-4 (IL-4), interleukin-10 (IL-10), and interleukin-13 (IL-13), tend to inhibit IDO1 expression or negatively modulate the activation of the KP, thereby supporting an anti-inflammatory response.

An imbalance between TH1 and TH2 cytokines associated with an altered hypothalamic–pituitary–adrenal (HPA) axis and elevated circulating damage-associated molecular patterns (DAMPs) can disrupt this delicate metabolic pathway, favoring TH1 polarization and indirectly influencing the risk of developing neurological or psychiatric disorders via increased oxidative stress and glutamatergic cytotoxicity. Thus, the interaction and crosstalk between the adaptive immune system and Trp metabolism via the KP represents a central and critical equilibrium point governing inflammation, mood regulation, fatigue, and neurocognitive health [18]. Specifically, Lotfi et al. (2018) showed that ATP significantly upregulated IDO, enhancing conversion of tryptophan to kynurenine in bone marrow-derived mesenchymal stem cells and that conditioned medium derived from these, strongly suppressed Th1-type lymphocyte proliferation [19]. Blocking ATP with apyrase or inhibiting P2X7 receptors prevented this effect, confirming ATP-driven signaling and identifying ATP as a key DAMP that can boost MSC-mediated immunosuppression through the kynurenine pathway [19].

The indole pathway leads to the transformation of Trp by gut-resident microbes such as Clostridium sporogenes, Lactobacillus reuteri, or Escherichia coli into indole derivatives, which act on the aryl hydrocarbon receptor (AhR) and pregnane X receptor (PXR), thereby modulating intestinal homeostasis, mucosal immunity, BBB integrity, and even neuroinflammatory responses via the gut–brain axis, as reviewed in [18,20].

## 3. KP Metabolites as Regulators of Neuroprotection and Excitotoxicity Across the BBB

The KP generates metabolites with either neuroprotective or neurotoxic properties that can cross the BBB and play a critical role in regulating mood, cognition, oxidative stress, and neuroinflammation, as reviewed in [17]. Patel et al. (2021) demonstrated using micro-dialysis assays in rat pre-frontal cortex that KYN crosses the BBB via large neutral amino acid transporter (LAT1), where it is converted into KYNA and transported back out of the brain via OAT1/3 and MRP-4 [21]. KYNA acts as an antagonist of N-methyl-D-aspartate receptor (NMDA) receptors by blocking the glycine binding site, as an antagonist of the α7 nicotinic acetylcholine receptor (α7 nAChR), as an agonist of the G protein–coupled receptor 35 (GPR35), and as a partial agonist of the AhR, thereby exerting neuroprotective, antiexcitotoxic, and anti-inflammatory effects respectively, details covered in a systematic review [22,23].

**Figure 2:**
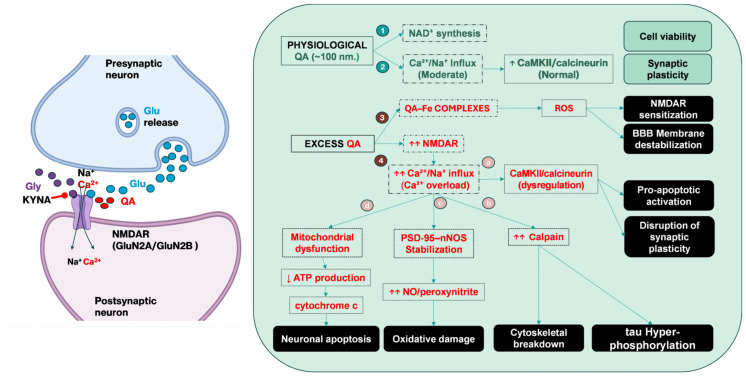
Dual role of QA in neuronal physiology and pathology through NMDA receptor modulation. Under physiological conditions (~100 nM), marked in green, QA contributes to neuronal survival by stimulating NAD^+^ synthesis, inducing moderate Ca^2+^/Na^+^ influx through NMDA receptors, and maintaining CaMKII/calcineurin activity, thereby supporting synaptic plasticity and cell viability. Benefic outcomes of QA in physiological conditions are presented in green boxes. In pathological states (excess QA, 150–1200 nM), marked in red, QA acts as a potent NMDA receptor agonist, causing Ca^2+^ overload, stabilization of the PSD-95–nNOS complex with excessive NO/peroxynitrite generation, and activation of calpains. In parallel, QA–Fe complexes promote ROS formation and lipid peroxidation, leading to NMDA receptor sensitization and BBB disruption. These mechanisms include mitochondrial dysfunction, ATP depletion, cytochrome c release, cytoskeletal breakdown, tau hyperphosphorylation, oxidative stress, and neuronal apoptosis, ultimately resulting in excitotoxicity and neurodegeneration. Black boxes illustrate the consequences of QA excess. (QA—Quinolinic acid, NMDAR—N-methyl-D-aspartate receptor, Glu—glutamate, Gly—glycine, KYNA—kynurenic acid, CaMKII—calcium/calmodulin-dependent protein kinase II, PSD-95—postsynaptic density protein 95, nNOS—neuronal nitric oxide synthase, NO—nitric oxide, ROS—reactive oxygen species, ATP—adenosine triphosphate, BBB—blood–brain barrier, ↑ increased effect, ↓ decreased effect). Created in BioRender. Tero-Vescan, A. (2025) https://BioRender.com/xt2b9qf (accessed on 2 November 2025).

In contrast, KYN metabolism through KMO leads to the production of QA, a potent NMDA receptor agonist that plays a dual, concentration-dependent role within the central nervous system (CNS) [24]. As early as 2001, Guillemin et al., observed high concentrations of KA in human cultured astrocytes due to a lack of endogenously produced kynurenine hydroxylase and concomitant with this was production of large amounts of potentially toxic QA within the brain if translatable in vivo [25]. At physiological levels (~100 nM), QA stimulates NAD^+^ production and promotes neural stem cell proliferation and differentiation, processes essential for hippocampal neurogenesis and cognitive resilience, whereas at elevated concentrations (150–1200 nM), QA shifts from a physiological modulator to a potent neurotoxin [26]. QA acts selectively on NMDA receptors containing GluN2A/GluN2B subunits and, to a lesser extent, on GluN2C/GluN2D, thereby explaining the increased vulnerability of the hippocampus, cortex, and striatum compared with the greater relative resistance of the cerebellum and spinal cord [27]. The dual role of QA in neuronal physiology and pathology through NMDA receptor modulation is described in Figure 2.

Under pathological conditions, in a series of studies on rodents, when QA was artificially elevated resulting in increased KA production, NMDA receptor overactivation induced excessive Ca^2+^ and Na^+^ influx, aberrantly activating calcium/calmodulin-dependent protein kinase II (CaMKII) and calcineurin, stabilizing the post synaptic density PSD-95–nNOS complex with overproduction of NO/peroxynitrite and oxidative stress, ultimately leading to stimulation of calpains and other proteases that resulted in disruption of the cytoskeleton [28,29]. When QA was infused into the right lateral ventricle of Wistar rats, impaired short-term memory and learning were also impaired concomitant with IHC identification of increased tau phosphorylation in the injected region [30]. These processes further drive mitochondrial dysfunction and neuronal apoptosis

Moreover, in Wistar rat brain homogenates, QA–iron complexes promoted the pathological generation of thiobarbituric reactive species (TBARS) and lipid peroxidation, which acted through the NMDA receptors supporting further the concept of KA-associated destabilization of brain ECM and BBB membranes, thereby exacerbating neuronal injury and tissue vulnerability [31,32].

The QA/KYNA ratio is considered a biomarker for assessing neuroinflammatory status, mood disturbances, and central fatigue, with its value being strongly influenced by immune activation and stress [33]. Elevated QA/KYNA ratios have been consistently reported in patients with MDD, bipolar depression, and chronic fatigue and are correlated with symptom severity, cognitive impairment, and the levels of circulating proinflammatory cytokines such as IFN-γ, IL-6, and TNF-α (see the next section for a more elaborate explanation) [24,34]. Neuroimaging studies further show that higher QA/KYNA ratios are associated with hippocampal volume loss and altered glutamate–glutamine cycling, linking biochemical imbalance to structural and functional brain changes [35]. Mechanistically, as defined earlier, this ratio reflects the opposing actions of quinolinic acid, a potent NMDA receptor agonist that promotes excitotoxicity and oxidative stress, and KA. Immune activation skews Trp metabolism toward the KMO-driven, QA-producing branch while suppressing astrocytic KAT activity and KYNA synthesis, thereby creating a proexcitotoxic environment. Consequently, the QA/KYNA ratio integrates immune and metabolic status into a single measurable index of neuroimmune imbalance. Normalization of this ratio following exercise training, anti-inflammatory therapy, or antidepressant treatment has been associated with improved mood and cognitive outcomes, indicating its value as a dynamic translational biomarker of neuroinflammation and treatment responsiveness in MDD and other neurodegenerative conditions. For example, significantly higher QA/KYN and lower KA/KYN ratios seen in patients with borderline personality disorder were reversed together with increased myokine IL-6 expression following acute cycling exercise routines and compared with controls indicating neuroprotective impact [4]. Whilst still at the experimental phase, ginsenosides (derivatives of ginseng) were recently shown to specifically target kyneurenine 3-monooxygenase using molecular docking and site-directed mutagenesis assays whilst most importantly, the authors went on to show that the ginsenoside metabolic compound localised in the thalamic neurones of the brain of cortisone-induced depressed mice, normalising the QA/KA ratios and alleviating the symptoms [36].

Several recent studies also indicated a potential role of KP dysregulation in Parkinson’s Disease (PD) progression, and in this light, Wilson et al. (2025, pre-print), showed that increased neuroexcitatory as defined by the QA/KA ratio in both plasma and CSF of 177 PD participants was associated with both peripheral and cerebral inflammation as well as vitamin B6 deficiency [37]. Furthermore, increased QA correlated with CSF tau expression and severity of both motor and non-motor PD clinical dysfunction indicating the possible importance of tracking KA metabolic pathways to monitor and predict clinical progression or outcomes [34,37].

In a recent multinational cross-sectional study including 353 persons with multiple sclerosis and 111 healthy controls, targeted LC-MS/MS profiling of serum KP metabolites followed by exploratory factor analysis revealed two distinct KP metabolite patterns, an inflammation-driven neurotoxic profile (NeuroTox), characterized by higher neopterin, KYN/Trp ratio, 3-HK, QA and an increased QA/KA ratio, and a neuroprotective profile (NeuroPro), characterized by higher KYNA, quinaldic acid, XA and picolinic acid together with an inverse loading of the QA/KA ratio. Greater NeuroTox and lower NeuroPro scores were associated with higher Expanded Disability Status Scale (EDSS) scores, older age, increased BMI and body fat percentage, whereas higher NeuroPro scores were linked to lower body fat percentage and superior cardiorespiratory fitness (relative $\overset{\cdot }{V}$O_2_ peak) [38].

## 4. Inflammatory Cytokine Networks Regulate IDO1 Expression and the QA/KYNA Balance in Neuroinflammation

Proinflammatory cytokines such as IFN-γ, IL-6, and TNF-α are potent inducers of IDO1, thereby promoting the conversion of Trp into KYN and subsequently into QA while simultaneously reducing Trp availability for 5-HT synthesis [39]. A review by Stone et al. (2022) confirmed that IFN-γ is the most powerful inducer of IDO1, whereas other proinflammatory stimuli, such as IL-6, IL-1β, and TNF-α, act primarily to amplify and sustain this signal [40]. IFN-γ is the principal inducer of IDO1 expression through activation of the Janus kinase/signal transducer and activator of transcription 1/interferon regulatory factor 1 (JAK/STAT1/IRF1) cascade. JAK phosphorylates STAT1, which dimerizes, translocates to the nucleus, and binds the Gamma-Activated Sequences (GAS) GAS-2 and GAS-3 promoter regions of the IDO1 gene, directly driving transcription. In parallel, IFN-γ induces IRF-1, which binds the ISRE-1 and ISRE-2 elements, further enhancing IDO1 activation. Complete STAT1 activation requires the phosphoinositide 3-kinase alpha (PI3Kα) pathway. Similar mechanisms have been reported for IFN-β, which upregulates IDO1 via STAT1/STAT2 and engages the IDO1–KYN–AhR metabolic circuit [41]. IFN-α/β are considered secondary inducers of IDO1, particularly in viral infections such as was shown recently in COVID-19 infection, where amplified IFN-γ–driven response and accelerating Trp depletion (and increased KA levels) occurred concomitantly with increased inflammatory marker expression such as IL-6, and these were associated with and correlated to higher mortality [42].

IL-6 alone is also not considered a primary inducer of IDO1 but acts as a modulator/amplifier of IDO activity, often by sustaining or enhancing IFN-γ–driven induction [43]. Similarly, TNF-α, IL-1β, IL-2, IL-8, IL-12, IL-17 and IL-18 function as costimulators by stabilizing IDO1 mRNA and maintaining its activity, thereby directing Trp metabolism toward the KYN pathway. In microglia, TNF-α further promotes IDO1 activity through NF-κB signaling, linking neuroinflammation with excitotoxicity via excessive QA formation, for example, using a mouse model of post-stroke depression, it was shown that blocking IDO1 with Xiangshao granules inhibited both ILs and TNF-α through NF-κB whilst increasing expression of 5HT and reversing anxiolytic behaviour in the animals [44,45].

Collectively, these findings emphasize that IDO1 activation is not only a direct response to IFN-γ but also a complex result of a cytokine milieu in which IL-6, TNF-α, IL-1β, and type I interferons synergistically regulate Trp catabolism and tilt the QA/KYNA balance toward neurotoxicity, oxidative stress, and mood dysregulation.

## 5. Exercise Drives Positive Remodeling of the KP

Physical exercise modulates systemic inflammation, increases KAT activity, and promotes the conversion of KYN into KYNA [7]. A study conducted in adults (males and females aged 25–65 years) subjected to daily supervised exercise every morning for four consecutive weeks demonstrated that physical activity significantly increased KAT activity, reduced circulating KYN (25%), and elevated KYNA (32%) concentrations in sweat. This finding also highlights sweat analysis as a noninvasive method capable of quantifying the type and intensity of exercise required to induce KYNA formation with neuroprotective effects [46]. Similarly, Brzezinska et al. [47] showed a significant increase in KYNA/KYN ratio concomitant with an increase in Trp in ischaemia pre-conditioned individuals following intense track interval running sessions compared with a non-exercising control cohort.

Consistent with the neuroprotective effects of exercise on the KP, a study using 36 Sprague–Dawley rats exposed to 4 weeks of voluntary wheel running markedly reduced hippocampal QA and KMO expression while increasing KA and KAT levels, thereby shifting KP metabolism toward its neuroprotective branch and improving depressive-like behavior [48]. A study on 18 young Thoroughbred race horses subjected to high-intensity exercise tests, plasma analyses collected at rest and 10 and 60 min post-effort revealed that intense physical exertion increased lactate, KYN, XA and nicotinamide levels while decreasing Trp, with exercise-induced rises in lactate correlating positively with 3-HK, XA and nicotinamide, indicating an acute, intensity-dependent modulation of the KP in equine skeletal muscle with no adverse health effects expected [49]. This was confirmed in a murine model where exhaustive exercise resulted in reduced KAT1 and increased KAT3 in skeletal muscle concomitant with increased KYN in the hippocampal region of the brain indicating a shift towards neurotoxic phenotype [50]. In vitro using C2C12 myoblasts data went on to show the involvement of a novel mechanism whereby inhibition of nuclear receptor subfamily 1 group D member 1 (NR1D1), which is a critical mediator of muscle oxidative stress, was sufficient to normalize the KYN pathway and block neurotoxicity.

Another preclinical study in young DBA/2J mdx mice with a severe Duchenne muscular dystrophy model, KP is markedly dysregulated, with reduced circulating KYNA, a lower KYNA/KYN ratio, decreased skeletal-muscle PGC-1α and KAT1, and elevated TNF-α expression, alterations that correlate with anxiety-like behaviour and suggest that impaired muscle health can shift the KP away from its neuroprotective branch [51].

In a single-blind randomized trial of 69 people with multiple sclerosis (MS) (EDSS 3.0–6.0) undergoing 3 weeks of inpatient rehab, participants were assigned to cycling-based high-intensity interval training (HIIT) versus moderate continuous training (MCT). Plasma was sampled at baseline, immediately after the first session, 3 h postintervention, and postintervention; Trp, KYN, KYNA, and QA were quantified by LC–MS/MS, with analyses at baseline and adjusted for IL-6. HIIT acutely increased KYNA and reduced the QA/KYNA ratio, and after 3 weeks, the KP was shifted toward KYNA more with HIIT than with MCT, which is consistent with neuroprotective rerouting [52]. Furthermore, Hinkley JM et al. (2023) investigated 44 men and women divided into three groups according to age and physical activity status (young active/trained, older active/trained, and older sedentary) [7]. Their results demonstrated that in skeletal muscle, the KP is influenced by both age and activity level: elderly individuals accumulated KYN, whereas physically active participants presented increased levels of neuroprotective downstream metabolites, such as KYNA and NAD^+^, suggesting that exercise contributes to the maintenance of a healthy aging phenotype [7]. Comparing the effects of psychological (Trier Social Stress Test) and physical (Wingate test) stress in 35 healthy young men (aged 24.09 ± 3.39 years) showed that only acute physical stress elicited a markedly stronger activation of the KP, shifting metabolism toward KYNA and inducing greater cytokine reactivity [53].

Although exercise generally promotes a favorable shift in the KP, a study in 7 semi-professional long-distance runners showed lower XA and KYNA/KYN ratio and increased Trp degradation after 14 days of remote ischemic preconditioning, demonstrating that certain training-related stimuli can instead attenuate the neuroprotective branch of the KP, highlighting that exercise-induced metabolic effects are highly modality- and context-dependent [47]. In a placebo-controlled SIIT study in 20 elderly men (~65 years) performing 4–6 × 30-s cycling sprints, daily supplementation with vitamins C (1 g/day) and E (235 mg/day) abolished the beneficial exercise-induced effects on the KP, specifically the reductions in plasma QA, the increase in the KYNA/QA ratio, and the training-induced upregulation of muscle KAT demonstrating the role of physiological ROS as inducers of PGC-1α [54]. Most recently, a 2025 systematic review of 13 experimental and clinical studies including 592 participants further confirmed that physical activity, particularly HIIT, reduced circulating KYN and enhanced KAT-mediated conversion into KYNA in both healthy individuals and patients with chronic diseases, such as MS patients and cancer survivors. Collectively, these findings establish exercise as a potential nonpharmacological strategy capable of restoring KP homeostasis; improving cognitive resilience, mood, and fatigue resistance; and potentially complementing pharmacological therapies in controlling inflammation-driven disorders [23]. HIIT superior effects on neuroprotective branch of KP are demonstrated by a recent randomized trial involving 69 individuals with multiple sclerosis subjected to a 3-week HIIT, compared to MCT [55]. Together, these data demonstrate that exercise can significantly modify KYN metabolism toward a neuroprotective phenotype; however, the underlying molecular mediators of this effect are largely driven by exercise-induced myokines and adipokines, which act as systemic regulators of the KP.

Beyond immune activation, peripheral metabolic tissues such as skeletal muscle and adipose tissue play a decisive role in determining KP homeostasis and its impact on mood regulation. Through distinct but interconnected signaling axes, exercise-induced myokines and adipokines influence KYN metabolism, thereby modulating the balance between neuroprotective and neurotoxic metabolites that affect brain function.

### 5.1. The Muscle–Brain Axis: Exercise-Induced Myokines and KYN Clearance

**Figure 3:**
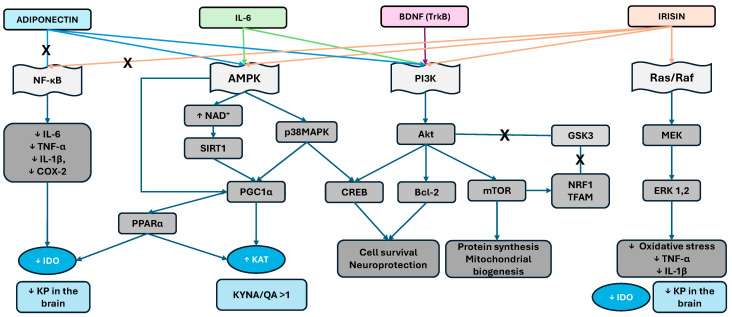
Schematic representation of the molecular pathways linking exercise-induced myokines (irisin, IL-6, and BDNF) and adipokines (adiponectin) to KP regulation and neuroprotection. Adiponectin inhibits NF-κB signaling via AMPK/SIRT1 activation, reducing the levels of proinflammatory cytokines (IL-6, TNF-α, IL-1β, and COX-2) and downregulating IDO, thereby limiting peripheral KYN accumulation. AMPK, which is activated by adiponectin, irisin and IL-6, stimulates PGC-1α and PPARα, which enhance KAT activity and favor the production of KYNA over QA. Through PI3K/Akt and Ras/Raf/MEK/ERK signaling, irisin and BDNF promote cell survival, neuroprotection, and mitochondrial biogenesis and reduce oxidative stress while also indirectly sustaining KAT activity. Crosstalk between AMPK, Akt, ERK1/2, and CREB integrates metabolic and neurotrophic signals, leading to increased cellular resilience, protein synthesis, and neuroprotection. Collectively, these pathways remodel the KP toward a neuroprotective profile (↑ KYNA/QA ratio) and reduce neurotoxic drive in the brain. (AMPK—AMP-activated protein kinase, Akt—Protein kinase B, BDNF—Brain-derived neurotrophic factor, COX-2—Cyclooxygenase-2, CREB—cAMP response element-binding protein, ERK1/2—Extracellular signal-regulated kinases ½, GSK3—Glycogen synthase kinase 3, IDO—Indoleamine 2,3-dioxygenase, IL-1β—Interleukin-1 beta, IL-6—Interleukin-6, IRF1—Interferon regulatory factor 1, KAT—Kynurenine aminotransferase, KP—Kynurenine pathway, KYNA—Kynurenic acid, mTOR—Mechanistic target of rapamycin, NF-κB—Nuclear factor kappa B, NRF1—Nuclear respiratory factor 1, PGC-1α—Peroxisome proliferator-activated receptor gamma coactivator 1-alpha, PI3K—Phosphatidylinositol-3-kinase, PPARα—Peroxisome proliferator-activated receptor alpha, QA—Quinolinic acid, Raf—Rapidly accelerated Fibrosarcoma kinase, Ras—RBat sarcoma oncogene family GTPase, SIRT1—Sirtuin 1, TFAM—Mitochondrial transcription factor A, TNF-α—Tumor necrosis factor alpha, TrkB—Tropomyosin receptor kinase B, down arrow ↓ indicate decrease, up arrow ↑ indicate increase, an X on a line indicates blocking).

During physical exercise, skeletal muscle serves as a key mediator of the KP, primarily through the release of myokines such as irisin, an activator of peroxisome proliferator-activated receptor gamma coactivator-1α (PGC-1α) (see Figure 3), which in turn induces KAT, which promotes the conversion of KYN into KYNA in peripheral tissues. By limiting the entry of KYN into the brain, the muscle–brain axis functions as a protective buffer against excitotoxicity, oxidative stress, and mood dysregulation [56,57].

A preclinical study in transgenic mck-PGC-1α1 mice (muscle creatine kinase–PGC-1α1) demonstrated that overexpression of the PGC-1α–PPAR–KAT axis in skeletal muscle modulated the KP and protected against depression-like behaviors induced by chronic mild stress induced by the forced swim test [58]. This and other similar works collectively establish the muscle–brain axis as a critical mediator of the antidepressant and neuroprotective effects of exercise. By enhancing peripheral KYN clearance and increasing KYNA production, exercise not only improves resilience to stress and mood disturbances but also may complement pharmacological therapies for inflammation-driven disorders.

IL-6, released acutely at levels up to 100-fold above baseline following intense or prolonged exercise, functions both as a metabolic regulator via AMPK and PI3K/Akt activation and as an immunomodulator by attenuating oxidative stress and glutamatergic excitotoxicity, thereby indirectly sustaining KAT activity through enhanced substrate utilization and reinforcement of anti-inflammatory signaling pathways [59]. Similarly, using a transgenic over-expressing PGC-1α mouse model of endurance exercise and RAW 264.7 macrophage cell line, Furrer et al. (2017) showed that BDNF was upregulated during endurance and HIIT, and although not directly enzymatic, it synergized with PGC-1α in skeletal muscle to reinforce neuroprotective adaptations, through M1 priming but M2 preference (anti-inflammatory) macrophage activation which associates with muscular protection and regeneration as well as improved mitochondrial biogenesis-and in the broader context, potential resilience against stress-induced mood disorders [60]. Additional myokines, such as apelin and fibroblast growth factor 21 (FGF21), may also modulate the KP indirectly through effects on mitochondrial bioenergetics, NAD^+^ metabolism, and systemic inflammation. For example, in a comparison of apelin knockout mice with wild-type, exposed to HIIT by treadmill running, KO mice showed abnormal defective cardiac and skeletal myotubular growth together with defective skeletal muscle oxidative capacity, insulin receptor substrate-1 expression and mitochondrial biogenesis. Since apelin secretion peaks during exercise, interval training or enhanced physical activity in general should support and facilitate peripheral KP activity at least in part through this mechanism [61]. Similarly, FGF21, which is secreted by muscle and liver in response to exercise and metabolic stress, regulates glucose and lipid metabolism, activates SIRT1 and PGC-1α, and enhances NAD^+^ turnover, thereby indirectly promoting KAT activity and favoring the conversion of KYN into KYNA [62]. Both apelin and FGF21 also exert systemic anti-inflammatory effects by attenuating NF-κB signaling and reducing proinflammatory cytokine production. In this regard, apelin significantly improved post-stroke neurological function, vascular integrity/BBB function and microglial M2 polarization via SIRT1/NF-κB signalling pathways in a mouse model of middle cerebral arterial occlusion-reperfusion injury [63]. Similarly, administration of FGF-21 to diabetic ageing mice over a period of 6 months, protected hippocampal neurones from karyopyknosis and oedema, suppressed aggregation of tau and β-amyloid and reduced signs of oxidative stress via inhibition of NF-κB signalling pathways [63,64].

However, while clinical data indicate that these myokines may contribute to improved KYN clearance and neuroprotection, their precise mechanistic links to KP modulation in humans remain to be fully elucidated and warrant further translational investigation [65].

### 5.2. Importance of the Adipose Tissue Axis in Exercise and Mood: IDO1 Activation, Adiponectin Protection, and Leptin Complexity

In obesity and metabolic disorders, dysfunctional adipocytes upregulate IDO1, thereby becoming an additional peripheral source of KYN. In a study by Huang et al. (2022), the level of plasma bound circulating KYN correlated with BMI in overweight females in a cohort of 735 patients [66]. The authors went on to study IDO1-over expressing mice, showing that KYN was concomitantly over-expressed, primarily through adipocytes of the white fat tissue (rather than skeletal muscle), and subsequent depletion of IDO in mature adipocytes cell line 3T3L1, stabilised and reduced lipogenesis through reduction in STAT-3 expression amongst other signalling molecules. This aberrant activation amplifies systemic KYN levels, promotes chronic low-grade inflammation, and facilitates the accumulation of neurotoxic downstream metabolites such as QA, linking adipose tissue dysfunction to KP-mediated neurotoxicity and mood disturbances [66]. Adiponectin exerts anti-inflammatory and metabolic effects by suppressing NF-κB signaling and downregulating IDO1 expression while simultaneously enhancing mitochondrial biogenesis and fatty acid oxidation via the AMPK/SIRT1/PGC-1α axis, indirectly promoting KAT activity and shifting metabolism toward the production of neuroprotective KYNA [67].

Although recent findings suggest that exercise-induced increases in circulating adiponectin may reduce the neurotoxic load in individuals with obesity-related depression, direct evidence linking adiponectin to improved peripheral KYN clearance in humans remains limited and should be the subject of further investigation [68]. Synthetic adiponectin receptor agonists such as AdipoRon mimic these effects, lowering systemic inflammation and restoring Trp homeostasis in preclinical models. For example, treatment with AdipoRon successfully perturbed anxious, stress and depressive symptoms in an animal model of chronic sleep restriction. These effects, demonstrated in behavioural open field and ‘maze’ studies, were mediated through reduction in expression of the key pro-inflammatory protein IL-1β and associated signalling molecules through NF-κB, as well as modulation of corticosterone expression [69,70].

Leptin is involved in neuroimmune interactions that link obesity, inflammation, and mood disorders, as it stimulates the release of proinflammatory cytokines such as IL-6, TNF-α, and IFN-γ, which are well-established activators of IDO1, thereby increasing the degradation of Trp through the KP [71].

Although clinical studies have correlated higher KYN/Trp ratios, a proxy for IDO1 activation, with depressive symptom burden in patients with mood disorders, the evidence remains controversial when leptin signaling is considered: some human data show elevated leptin in MDD, which is consistent with inflammation-linked hyperleptinemia and increased IL-6/TNF-α signaling that can activate IDO1, whereas other reports (including bipolar disorder (BD) cohorts) describe lower leptin or state-dependent variation. Together, these discrepancies underscore the complexity of the leptin–cytokine–IDO1 axis in KP dysregulation [13,72]. A 2023 study conducted on patients with major depressive disorder (MDD), stratified into groups with high and low CRP levels and compared with healthy controls, showed that both MDD groups exhibited significantly higher leptin concentrations than controls. In MDD participants, elevated leptin levels were associated with reduced reward-anticipation–related activation in the left insula and dorsolateral putamen, whereas in healthy individuals leptin correlated positively with activation in these same regions. These findings suggest that MDD is characterized by an altered immuno-metabolic signature, with hyperleptinemia acting through inflammatory pathways distinct from CRP [73].

In the future, restoring leptin sensitivity through exercise, caloric restriction, or pharmacological sensitizers could represent an innovative strategy to normalize KP activity, rebalance the KYNA/QA ratio, and reduce vulnerability to mood disturbances.

## 6. The KP in Depression and BD: Neuroprotective Deficits and Neurotoxic Shifts

The KP plays a pivotal role in the pathophysiology of mood disorders, including MDD and BD. Reduced plasma levels of L-Trp was shown in many studies linked to MDD with a meta-analysis in 2014 showing mean plasma levels from a total of over 1500 patients being significantly decreased particularly in unmedicated patients [74]. Yun et al. (2025) showed in a cohort of 70 patients that depression, anxiety and pain associated with depression were correlated with the Trp/KYN ratio measured using mass spectrometry [75], whilst within a small cohort of MDD patients (n = 31), KYN levels in the cerebrospinal fluid (CSF), also associated with Trp/KYN ratio and correlating with the inflammatory biomarker neopterin over-expression also correlated with suicide attempts [76]. The overwhelming data therefore points to KYN, and increased KYN/Trp ratio, as strongly associated with depression, with elevated AA as an indicator of disease severity and CSF levels in MDD showing compartmentalized regulation and potential state dependence (depression vs. remission) One of the main mechanisms linking the KP to MDD is immune activation. Proinflammatory cytokines such as IFN-γ, IL-6, and TNF-α activate IDO and TDO, thereby diverting Trp away from 5-HT synthesis toward the KP, whereas the activation of KMO favors the neurotoxic branch of the pathway [13]. This immune–metabolic shift reduces central 5-HT availability and promotes the accumulation of neuroactive KP metabolites [77]. A recent meta analysis and systematic review provided evidence of specific cytokine alterations that appear to be state dependent, with primarily IL-6 and TNF-α consistently elevated during manic and depressive episodes. [78]. For example, manic depressive patients with BD (cohort of n = 130) showed higher expression of sIL-2R/6R and sTNFR1 when in the hypomanic euthymic state, measured by YMES and MADRE, correlating also with the length of the illness [79], and suggesting a link to Th2-skewed immune response in acute episodes. [78]. For example, manic depressive patients with BD (cohort of n = 130) showed higher expression of sIL-2R/6R and sTNFR1 when in the hypomanic euthymic state, measured by YMES and MADRE, correlating also with the length of the illness [79], and suggesting a link to Th2-skewed immune response in acute episodes.

C-reactive protein (CRP), an acute-phase marker induced by IL-6, normally does not cross the BBB; however, in the presence of inflammation, it can enter the CNS, primarily residing in its monomeric, tissue insoluble form (mCRP) [80]. A 2024 review reported higher CRP levels in both MDD patients and BD patients than in healthy controls. Notably, the increases were more pronounced and phase-dependent in BD patients, with CRP concentrations rising during depressive and manic episodes and normalizing in remission. In contrast, elevations in MDD patients were more modest but correlated with depressive symptom severity and central inflammatory markers. These findings suggest a fluctuating yet stronger involvement of the immune–inflammatory response system in BD patients than in MDD patients and the possibility that modulation of active markers such as CRP could modify or support therapeutic interventions in MDD [80,81,82]. Liu et al. (2026) reported circulating CRP levels were associated with higher baseline depressive symptom severity in patients with MDD in a cohort of 55 individuals, whilst IL-6 reduction was strongly corelated to depressive symptom relief [83]. Another mechanism implicated in the relationship between the KP and mood disorders is the imbalance between neuroactive metabolites, specifically the reduction in KYNA and the KYNA/QA and KYNA/3-HK ratios, which reflects a pathological shift toward excitotoxic and proinflammatory metabolites that act on NMDA receptors and the α7 nAChR [84]. Excess QA, produced by activated microglia and acting as a potent NMDA receptor agonist, drives overshooting glutamatergic signaling, leading to neuronal excitotoxicity and disrupting the physiological balance with neuroprotective KYNA. This imbalance contributes to vulnerability to depression and suicidality [85]. Furthermore, sex-specific differences have been reported in the literature: lower KYNA levels and reduced KYNA/QA ratios in the anterior cingulate cortex of women with MDD who die by suicide, compared with both controls and their male counterparts, underscore the contribution of glial-mediated neurotoxicity to suicidality risk [86].

KYNA has been proposed as a potential predictive biomarker of antidepressant efficacy. In patients with MDD, lower baseline KYNA levels are associated with a better therapeutic response to serotonergic antidepressants, likely through reduced glutamatergic inhibition and restoration of excitatory–inhibitory balance [87].

A recent study demonstrated that patients with MDD exhibit gene expression dysregulation of key KP enzymes, including KAT1, KYNU, and IDO, compared with healthy controls. These findings suggest altered transcriptional regulation of the KP in depression, potentially contributing to imbalances in neuroactive metabolites and reinforcing the role of KP dysregulation in mood disorder pathophysiology [88].

Taken together, these findings suggest that mood disorders are characterized by a consistent pattern of KP dysregulation, involving both reduced neuroprotective KYNA signaling and increased production of neurotoxic metabolites such as 3-HK and QA. This imbalance contributes to excitotoxicity, microglial hyperactivation, and altered neurotransmitter dynamics, thereby linking immune activation with mood disturbance and suicidal vulnerability.

### KP Dysregulation in Alzheimer’s Disease and Dementia Future Considerations

KP alterations in AD mirror the immune–metabolic shifts observed in mood disorders, with Trp depletion and a higher KYN/Trp ratio indicating peripheral KP activation, alongside a central–peripheral dissociation (CSF KYNA ↑ despite KYNA ↓ in blood). A 2023 systematic review (22 studies; n = 1356 AD vs. controls) reported reduced Trp and elevated KYN/Trp peripherally and higher CSF KYNA, underscoring compartmentalized regulation that likely varies with disease stage and neuroinflammatory tone [89].

In addition to being involved in flux, AhR signaling triggered by KP ligands such as KYN modulates microglial states. While physiologic KYN–AhR can restrain inflammation, chronic elevation of KYN under sustained immune activation may bias microglia toward neurotoxic phenotypes, converging with QA-mediated NMDA overstimulation and oxidative stress to amplify synaptic vulnerability [90,91].

In a mouse model of AD induced by intracerebroventricular Aβ1-42 administration, eight weeks of swimming training prevented memory deficits, anxiety- and depressive-like behaviors, and neuroinflammation, while blocking Aβ1-42–induced increases in IDO activity, TRP and KYN levels, and the KYN/TRP ratio in the prefrontal cortex and hippocampus, demonstrating that exercise attenuates AD-related neuropathology partly through suppressing the neurotoxic activation of the KP [92].

A 2025 meta-analysis further revealed central KP overactivation with long-term Trp depletion, reduced blood KYNA, XA, and 3-HAA, and brain accumulation of neuroactive metabolites, patterns consistent with a shift toward the KMO → 3-HK/QA (neurotoxic) branch and away from the KAT → KYNA (neuroprotective) pathways [93]. Nevertheless, aside from the robust finding of low circulating Trp across Alzheimer’s disease (AD), Parkinson’s disease (PD), and Huntington’s disease (HD), between-study heterogeneity for other metabolites remains high, suggesting the need for longitudinal, multimodal biomarker designs to define disease-specific vs. pan-neurodegenerative signatures [94]. This implies that, as in MDD/BD, AD is characterized by reduced KYNA signaling and excess 3-HK/QA, linking immune activation to excitotoxicity and microglial dysregulation. Therapeutically, strategies aiming to rebalance KYNA/QA (e.g., KMO inhibition, exercise/HIIT protocols that shift KP toward KYNA, or AhR-aware anti-inflammatories) merit prospective testing for neurocognitive resilience.

## 7. Intervention Strategies: Exercise and Exercise Mimetics

**Figure 4:**
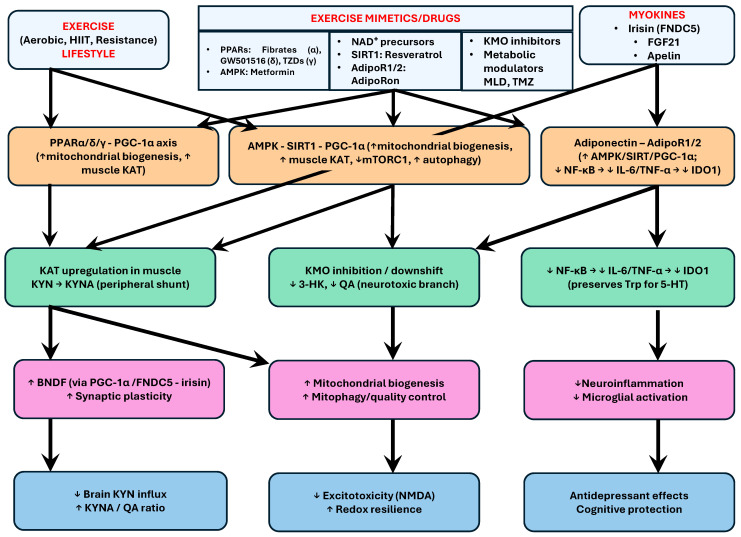
Overlapping therapeutic signaling map linking exercise, exercise mimetics and the KP in mood disorders. The schematic integrates lifestyle and pharmacological levers that converge on three central nodes, PPAR–PGC-1α, AMPK–SIRT1, and adiponectin–AdipoR, to rebalance kynurenine pathway (KP) flux, increase mitochondrial quality control, and dampen neuroinflammation. Exercise (aerobic, HIIT, resistance) and myokines (irisin, FGF21, apelin) activate PGC-1α and AMPK/SIRT1, whereas exercise mimetics (e.g., fibrates, GW501516, TZDs, metformin, NAD^+^ precursors, resveratrol, AdipoRon, KMO inhibitors, meldonium, trimetazidine) target the same hubs. Downstream, PPAR/PGC-1α and AMPK/SIRT1 upregulate KAT in skeletal muscle, promoting the peripheral shunt of KYN → KYNA, thereby lowering brain KYN influx. In parallel, KMO inhibition/downshift reduces the formation of 3-HK and QA (neurotoxic branch). Adiponectin → AdipoR1/2 signaling activates AMPK/SIRT1/PGC-1α and suppresses NF-κB–driven cytokines (IL-6, TNF-α), which decreases IDO1 activity and preserves Trp for 5-HT synthesis. These intersecting routes increase the KYNA/QA ratio, support mitochondrial biogenesis and mitophagy (via AMPK-mediated mTORC1 inhibition), increase BDNF (via PGC-1α/FNDC5–irisin), and attenuate microglial activation, collectively reducing NMDA-mediated excitotoxicity, improving redox resilience, and yielding antidepressant and cognitive-protective effects. (Down arrow ↓ indicate decrease, up arrow ↑ indicate increase).

Convergent mechanisms involving the PPAR–PGC-1α–KAT axis, the AMPK/SIRT1 pathway, adiponectin signaling, KMO/KAT modulation, and the irisin/FGF21/apelin myokine axis, exercise and its pharmacological mimetics collectively regulate KP homeostasis, mitochondrial biogenesis, and neuroimmune responses, thereby offering complementary and potentially synergistic strategies for alleviating depressive symptoms and preventing cognitive decline in MDD patients. Figure 4 illustrates the convergent molecular landscape through which exercise and pharmacological exercise-mimetics modulate the KP and related signaling cascades implicated in mood regulation. Table 1 summarizes the principal exercise modalities evaluated in mood disorders. A possible intervention with the potential to normalize the QA/KYNA ratio, restore Trp homeostasis, and mitigate inflammation-driven neuropsychiatric risk consisting of exercise modalities and medications is shown in Appendix A.

### 7.1. Potential Exercise Routes and Drug Interventions Targeting the PPAR–PGC-1α–KYN Axis in MDD Patients

Aerobic training is considered the first-line nonpharmacologic intervention for MDD and cognitive fatigue, a role that has been consistently recognized across numerous studies [95]. A 2023 meta-analysis of more than 200 randomized controlled trials confirmed that exercise produces sex, age, and exercise type moderate antidepressant effects, with walking/jogging or yoga being more effective in men or older adults and strength training being more efficient in women and younger adults [5]. Similarly, a systematic review and meta-analysis of nine randomized controlled trials including 678 adults with MDD not receiving antidepressants reported moderate and mild effects of exercise on this disorder, but the conclusion was that there remains uncertainty regarding the optimal type, intensity, duration, and frequency of exercise that may be most effective [96]. PPARα/δ → PGC-1α → KAT → KYNA is the mechanistic pathway that explains how exercise can reduce depressive symptoms. During aerobic or resistance training, muscle PPARα/δ and its coactivator PGC-1α are upregulated. This program induces KAT enzymes in skeletal muscle, which convert circulating KYN into KYNA. KYNA does not cross the BBB well, so this peripheral “shunt” lowers brain KYN influx, reducing the production of neurotoxic metabolites (3-HK/QA) and easing glutamatergic/excitotoxic stress [97].

Although fibrates are not commonly prescribed for depression, they may represent a valuable alternative in patients with depression comorbid with cardiovascular or metabolic diseases, as they exert pleiotropic neuroprotective actions by stimulating mitochondrial pro-survival factors (PGC-1α, TFAM), inhibiting astrocytic NF-κB, upregulating PPARα and Glial cell line–Derived Neurotrophic Factor (GDNF) secretion to support dopaminergic neurons, reducing amyloid plaque burden and glial activation, and increasing hippocampal BDNF production, thereby counteracting depressive symptoms and cognitive decline [98]. Likewise, exercise mimetics such as GW501516 (PPARδ agonist) and thiazolidinedione (PPARγ agonists) activate the PGC-1α–PPAR axis and can indirectly influence the KP; however, despite these mechanistic effects, their clinical use in mood disorders remains experimental, with potential relevance only in depression associated with cardiometabolic conditions [99,100].

### 7.2. Potential Exercise Routes and Drug Interventions Targeting the AMPK/SIRT1 Axis in MDD

AMPK is activated during strength or resistance training by the increased AMP/ATP ratio and stimulates SIRT1, an NAD^+^-dependent deacetylase, thereby reinforcing metabolic adaptations by deacetylating and activating PGC-1α, the master regulator of mitochondrial biogenesis [101]. In skeletal muscle, the AMPK/SIRT1/PGC-1α pathway also promotes the conversion of KYN to KYNA by upregulating KATs, thereby reducing neurotoxic KYN influx into the brain and buffering against mood disturbances. In adipose tissue, SIRT1 activation attenuates NF-κB signaling and inflammatory cytokine release (e.g., IL-6 and TNF-α), lowering IDO1 activity and preserving Trp availability for 5-HT synthesis [102,103]. For example, in the study of Agudelo et al. (2019), skeletal-muscle–specific PGC-1α1 overexpression in male C57BL/6J mice markedly increased KAT enzyme expression and the conversion of KYN to KYNA, elevated intramuscular glutamate, aspartate and malate levels, and enhanced mitochondrial respiration, while acute intraperitoneal KYN administration (2.5 mg/kg) further amplified energy production, demonstrating experimentally how exercise-responsive regulators such as PGC-1α1 (via AMPK/SIRT1 pathways) can divert peripheral KYN metabolism away from neurotoxic pathways and support metabolic–immune resilience [104].

Beyond mitochondrial biogenesis, AMPK–SIRT1 signaling also interfaces with additional molecular pathways relevant to mood regulation. For example, AMPK can inhibit mTORC1, thereby promoting autophagy and cellular quality control, processes that are impaired in depression and neurodegeneration [105]. SIRT1, through the deacetylation of FOXO3a and PGC-1α, enhances antioxidant defenses and stress resilience while also influencing circadian rhythm and synaptic plasticity [106]. AMPK/SIRT1 activation also facilitates BDNF expression, either directly or through PGC-1α/FNDC5–irisin signaling, providing an additional neurotrophic mechanism linking resistance exercise to antidepressant effects [107].

These molecular adaptations of the AMPK/SIRT1/PGC-1α axis may provide a mechanistic basis for the antidepressant effects of resistance training. Such effects are particularly relevant in individuals with metabolic vulnerability, such as those with obesity, type 2 diabetes, or sarcopenia, where systemic low-grade inflammation, impaired mitochondrial biogenesis, and altered Trp metabolism via the KP contribute to mood disturbances.

A preclinical study on 42 male C57BL/6J mice assigned to a control diet, high-fat diet (HFD), or HFD plus voluntary wheel running (HFD + VWR) for 10 weeks showed that long-term voluntary exercise selectively activates the PGC-1α arm of the AMPK–SIRT1–PGC-1α axis in a fiber-type–dependent manner, contributing to improved metabolic outcomes in HFD-induced obese mice [108].

Importantly, emerging evidence suggests that women and younger adults may experience stronger antidepressant effects from resistance training, likely due to sex- and age-specific immune–metabolic profiles marked by heightened inflammatory reactivity and increased vulnerability to mitochondrial dysfunction, as demonstrated in the randomized controlled trial by O’Sullivan, where 55 young adults (26 ± 5 years; 36 female) completed eight weeks of guidelines-based resistance exercise training twice weekly and exhibited clinically meaningful reductions in depressive symptoms. [109]. We suggest that pharmacological exercise mimetics such as metformin (AMPK activator), NAD^+^ precursors such as nicotinamide riboside or NMN (SIRT1 cofactors), and resveratrol (SIRT1 activator) could complement resistance training by enhancing AMPK/SIRT1/PGC-1α signaling. These strategies may amplify KYN clearance, increase mitochondrial biogenesis, and attenuate neuroinflammation, offering a combined therapeutic framework for patients in whom physical exercise is limited owing to disability, frailty, or comorbid disease [110,111,112].

In addition to classical exercise mimetics, metabolic modulators such as meldonium and trimetazidine may also contribute to the therapeutic potential of the AMPK/SIRT1/PGC-1α axis in MDD [113,114]. Meldonium, by inhibiting carnitine-dependent fatty acid transport, shifts energy metabolism toward glucose utilization, thereby increasing mitochondrial efficiency and reducing oxidative stress while also promoting endothelial protection and neurotrophic support [115]. A preclinical study conducted in rats, which received meldonium either intragastrically (i.g.) at doses ranging from 25–100 mg/kg or intravenously (i.v.) at a fixed dose prior to exposure to a simulated acute high-altitude environment, demonstrated that meldonium modulates key metabolic pathways, particularly influencing the levels of succinic acid and 3-hydroxypropionic acid—metabolites associated with mitochondrial energy turnover and biochemical adaptations to hypoxia [116].

Similarly, trimetazidine, a partial fatty acid oxidation inhibitor, improves ATP production under conditions of metabolic stress and preserves mitochondrial function, with additional evidence for attenuating neuroinflammation and oxidative damage [117]. In an experimental model using 24 adult male Swiss white mice pretreated with trimetazidine (50 mg/kg, i.p.) prior to the induction of acute hypoxic injury, trimetazidine demonstrated robust cardioprotective effects, largely through the activation of the AMPK–Nrf2 signaling axis in endotoxemic conditions. [118]. Although primarily prescribed for cardiometabolic disorders, these agents may indirectly influence KYN metabolism and neuroimmune regulation, placing them among promising adjunctive strategies for depression, particularly in patients with comorbid cardiovascular or metabolic disease.

Among the molecular pathways through which exercise exerts antidepressant effects, the AMPK/SIRT1 axis emerges as a central hub, coordinating mitochondrial biogenesis, KYN detoxification, and NAD^+^-dependent metabolic resilience. By linking energy sensing with neuroimmune regulation, this pathway provides a unifying mechanism that complements the effects of aerobic and resistance training while offering new opportunities for pharmacological exercise mimetics [109].

### 7.3. Targeting Adiponectin Pathways: Exercise and Drug Strategies to Modulate IDO1 and KYN Metabolism

Adiponectin exerts key anti-inflammatory, metabolic, and neuroprotective effects with high implications in the context of mood regulation. Exercise, particularly aerobic and resistance training, consistently elevates circulating adiponectin levels, contributing to improved metabolic and inflammatory profiles [119,120].

A recent meta-analysis in adults with prediabetes and type 2 diabetes reported significant increases in adiponectin and concomitant reductions in leptin following structured exercise interventions, highlighting its role as a systemic mediator of exercise benefits [121]. Mechanistically, adiponectin suppresses NF-κB activity and downstream proinflammatory cytokine release (e.g., IL-6 and TNF-α), indirectly downregulating IDO1, thereby limiting excessive Trp catabolism into neurotoxic KYN metabolites and preserving substrate availability for 5-HT synthesis [122]. Simultaneously, adiponectin signaling through AdipoR1 activates the AMPK/SIRT1/PGC-1α axis, enhancing mitochondrial biogenesis and fatty acid oxidation while promoting KAT activity, ultimately shifting KYN metabolism toward the production of KYNA, a neuroprotective metabolite [123].

Preclinical data further suggest that pharmacological mimetics of adiponectin signaling may hold therapeutic promise. AdipoRon, a synthetic AdipoR1/R2 agonist, alleviated depression-like behavior in diabetic and stress-induced rodent models while also reducing neuroinflammation and improving hippocampal plasticity [70]. Importantly, intranasal administration of AdipoRon bypasses systemic barriers and confers robust anxiolytic and antidepressant-like effects, supporting translational feasibility [124]. These findings provide proof-of-concept for the use of adiponectin receptor agonists as potential adjunctive interventions in depression, particularly in patients with comorbid metabolic dysfunction.

Taken together, the dual role of adiponectin in reducing NF-κB/IDO1 signaling and activating the mitochondrial/PGC-1α pathways underscores its position as a systemic regulator of KP homeostasis. Lifestyle strategies that increase adiponectin secretion (aerobic training, resistance training, caloric restriction, and weight loss) and pharmacological interventions (AdipoRon or related receptor agonists) could therefore complement classical antidepressant strategies by directly targeting the metabolic–immune interface of mood disorders [125].

KMO is another potential therapeutic target of the KP, as it catalyzes the conversion of KYN into 3-HK, a precursor of QA, both of which are neurotoxic metabolites implicated in oxidative stress and glutamatergic excitotoxicity. Overactivation of KMO has been linked to MDD and neurodegeneration, where an imbalance between neurotoxic QA and neuroprotective KYNA contributes to mood dysregulation and cognitive impairment [126].

Like exercise, activation of KAT, pharmacological KMO inhibitors, such as Ro 61-8048 and novel brain-penetrant analogs, shift KP metabolism toward KYNA, thereby reducing the neurotoxic load and preserving neurotransmitter balance In a C57BL/6 mouse model of NAFLD induced by a high-fat diet combined with fructose–sucrose supplementation, pharmacological inhibition of KMO with RO 61-8048 markedly reduced hepatic steatosis, inflammation, lipid peroxidation, and apoptosis, lowering ALT, AST, TC, LDL-C, and pro-inflammatory cytokines, while mechanistic in vitro studies in FFA-challenged AML12 hepatocytes confirmed that RO 61-8048 attenuates lipid accumulation, ROS generation, and cell death via SP3-mediated regulation of the KMO promoter along the Trp-KYN pathway [127]. Thus, KMO inhibition and exercise converge mechanistically on the goal of lowering brain KYN influx and increasing KYNA availability through complementary routes such as enzyme blockade versus peripheral shunting.

Preclinical studies suggest that combining exercise with KMO inhibition could synergistically enhance resilience to stress-induced depression by jointly reducing 3-HK/QA production and reinforcing KYNA-driven neuroprotection [128] In male C57BL/6J mice (10–16 weeks old) subjected to spared nerve injury (SNI), intracerebroventricular administration of the IL-1 receptor antagonist (40 ng/day) or the KMO inhibitor Ro 61-8048 (0.4 μg/day) on days 6–7 prevented the SNI-induced increase in hippocampal KMO mRNA and fully reversed depression-like behavior in the forced swim test, without affecting mechanical allodynia, demonstrating that neuronal KMO activation in the contralateral hippocampus is required for the development of depressive symptoms in this model [129].

However, clinical translation remains limited, with current trials focused primarily on neurodegenerative disorders, highlighting the need for further targeted investigations into the antidepressant potential of KMO inhibitors alone or as adjuncts to lifestyle-based interventions.

### 7.4. Irisin/FGF21/Apelin Interactions in Exercise and Pharmacological Modulation of Mood Disorders

Different types of exercise elicit distinct myokine responses that converge on the KP, mitochondrial biogenesis, and neuroimmune regulation. Irisin, derived from FNDC5 cleavage, is predominantly released during aerobic endurance and HIIT, where sustained muscle contraction and PGC-1α activation drive its secretion. Irisin links physical activity to BDNF upregulation in the hippocampus, enhancing synaptic plasticity, stress resilience, and neuroprotection against glutamatergic excitotoxicity [130]. Apelin is maximally secreted during acute bouts of aerobic training and HIIT, where it improves endothelial function, stimulates angiogenesis and mitochondrial bioenergetics, and reduces NF-κB–mediated inflammatory tone. Its release is also increased in individuals undergoing resistance training, where apelin supports glucose uptake and metabolic remodeling in muscle [131].

From a translational perspective, pharmacological analogs of irisin, FGF21 (PF-05231023) [16], and apelin (e.g., [Pyr^1^]apelin-13, apelin-17 analogs, and more stable peptide mimetics) [23] are under preclinical or early clinical investigation, with promising roles in metabolic and neurodegenerative disorders. While their direct application in depression remains experimental, their convergence on PGC-1α, BDNF, and mitochondrial pathways underscores potential synergy with exercise in restoring KP homeostasis and reducing the QA/KYNA imbalance observed in MDD. Future interventions may combine structured training modalities with pharmacological mimetics of these myokines, offering a dual approach to enhancing neuroplasticity, metabolic resilience, and mood regulation.

**Table 1:** Key studies on exercise modalities and effects on mood disorders.

Effort Type	Participants/Gender	Duration	Proposed Mechanisms	Key Outcomes	Ref.
Aerobic (walking, jogging, dancing, cycling)	60 participants (40–60 years old)/women	3×/week, 30–45 min, moderate intensity, 8–12 weeks	↑ PGC-1α and IL-6 in skeletal muscle → ↑ KATs → ↑ KYN → KYNA peripheric conversion; ↓ systemic KYN influx to brain.	Moderate reduction in depressive symptoms; high acceptability	[132]
	46 CHF patients with CHF (40 to 60 years old)/study group (18 males, 5 females) and control group (17 males, 6 females)	3 ×/week, moderate intensity, 12 weeks		Positive effects of MICAE on the depression status in patients with CHF.	[133]
	14 BPD patients/3 males 11 females	30 min (5-min warm-up, 21-min active phase, 4-min cool-down); sessions ≥ 48 h apart		A single session of aerobic exercise increased KYNA, KYNA/KYN, KYNA/QA, and IL-6 levels, suggesting shift to neuroprotective profile	[4]
HIIT/SIT	17 participants/women	6×/6–10-min sessions, 3–12 “all out” sprints of 5 s interspersed with low-intensity recovery of 30–45 s, 2 weeks	Acute IL-6 and myokine burst	Small–moderate symptom reduction; cardiorespiratory fitness gains, significant improvements for HAM-D21	[134]
	34 patients suffering from unipolar depression (age: 37.8)/25 females + 9 males	3×/week for 4 weeks, 25 × 30-s cycling bouts at 80% VO_2_max interspersed with 30-s rest	acutely ↑ KYNA and ↓ QA/KYNA ratio	Beneficial effects of short-term exercise regimes on reduction of depressive symptoms and cardiovascular risk	[135]
RET	32 subjects (age 60–84)/study group (5 male, 12 female) and control group (7 male, 8 female)	2–3×/week, 6–8 exercises, 2–3 sets, 8–12 reps, 50–80% 1RM, 8–12 weeks	↑ AMPK/SIRT1, ↓ IL-6, TNF-α ↑ BDNF levels	Significant reductions in depression; larger effects in older adults; independent of strength gains	[136]
	36 BCSs (63.2 ± 1.1 years)	submaximal effort tests, 6-min walk test, 3-m Timed Up-and-Go, 5-repetition chair stands, usual gait speed	↑ PGC-1α, ↑ KAT, ↑ KYNA, ↓ KYN into the brain	Reduced inflammation-related neurotoxicity.	[137]
Combined Training (Aerobic + RET)	34 patients T2D (age: 60.6 ± 6.3)/women	3×/week: 20–30 min aerobic + 20–30 min RET circuits, 8–12 weeks	↑ AMPK/SIRT1, mitochondrial biogenesis, fatigue resistance	Equal or superior outcomes vs. single modality; metabolic benefits (insulin sensitivity)	[137]
	62 adults with unipolar depression (age 18–65 years)/combined physical activity training (13 males, 18 females) and aerobic activity training (9 males, 22 females)	6–8 weeks of Nordic walking + resistance + Qigong, + progressive muscle relaxation/or supervised aerobic Nordic walking		Significantly reduced depressive severity on the BDI-II	[138]
	15 patients with BD/8 females + 7 males	12 weeks of moderate-intensity aerobic exercise + RET/3 times per week		Positive impact on muscle strength and body composition, improvement in depressive symptoms and inflammation markers	[139]
Mind–Body Interventions (Yoga, Tai Chi, Qigong)	32 adults (age 37 ±12 years)/25 women and 5 men	2× 90-min + 3× 30-min homework/week;	↓ IL-6/TNF-α proinflammatory cytokines, potential ↓ IDO1 drive	Significant reduction in depressive severity; higher remission rates in yoga; Tai Chi effective in older adults	[140]
	112 older adults (mean age ~ 67 years)/68 women + 44 men	120-min weekly Tai Chi sessions/10 weeks			[141]

PGC-1α—peroxisome proliferator-activated receptor gamma coactivator 1-alpha, KATs—kynurenine aminotransferases, KYN—kynurenine, KYNA—kynurenic acid, IL-6—interleukin-6, AMPK—adenosine monophosphate-activated protein kinase, SIRT1—sirtuin 1, RET—resilience-enhancing training, HIIT—high-intensity interval training, SIT—sprint interval training, TNF-α—tumor necrosis factor-alpha, QA—quinolinic acid, IDO—indoleamine 2,3-dioxygenase, CHF—congestive heart failure, MICAE—moderate-intensity continuous aerobic exercise, HAM-D21 scores—Hamilton depression rating scale of 21-itens, BCS—breast cancer survivors, BDI-II—Beck depression inventory—second edition, BPD—borderline personality disorder, ↓ down arrow means decrease, ↑ up arrow means increase.

## 8. Conclusions

KPs act as a central metabolic interface linking exercise, inflammation, neuroprotection, and mood regulation. This relationship is supported by clinical studies showing that aerobic training, resistance training, HIIT training, and mind–body practices differentially influence KP metabolites and cytokine regulation. Secondly, myokine–adipokine crosstalk during exercise, via the secretion of irisin, IL-6, apelin, adiponectin, and leptin, functions as a systemic regulator of KP homeostasis. Finally, neuroimmune signaling, through IDO1 and KAT activation, BDNF–CREB pathways, mitochondrial biogenesis, and neurotransmitter balance, represents a converging mechanism that explains exercise-induced mood enhancement and cognitive resilience.

Clearer guidelines are needed on how exercise should be prescribed in terms of type, frequency, and intensity, especially in psychiatric populations. Sweat- or blood-based KP biomarkers could provide real-time monitoring of therapeutic effects. Multiomics platforms (metabolomics, transcriptomics, proteomics) help explain why individuals respond differently to KP remodeling. Exercise may also work best when combined with pharmacological strategies such as leptin sensitizers, IDO1 inhibitors, or NAD^+^ boosters, potentially boosting its antidepressant and neuroprotective effects.

## Data Availability

No new data were created or analyzed in this study. Data sharing is not applicable to this article.

## Abbreviations

The following abbreviations are used in this manuscript:

3-HAA	3-hydroxyanthranilic acid
3-HK	3-hydroxykynurenine
5-HIAA	5-hydroxyindoleacetic acid
5-HT	Serotonin
5-HTP	5-hydroxytryptophan
AA	Anthranilic acid
AADC	Aromatic L-amino acid decarboxylase
AANAT	N-acetyltransferase
AD	Alzheimer’s disease
AFMID	Arylformamidase
AhR	Aryl hydrocarbon receptor
Akt	Protein kinase B
Ala	Alanine
AMPK	AMP-activated protein kinase,
ASMT	N-acetylserotonin O-methyltransferase
ATP	Adenosine triphosphate
BBB	Blood–brain barrier
BD	Bipolar disorder
BDNF	Brain-Derived Neurotrophic Factor
CaMKII	Calcium/calmodulin-dependent protein kinase II
CNS	Central nervous system
COX-2	Cyclooxygenase-2
CREB	cAMP response element-binding protein
CRP	C-reactive protein
CSF	Cerebrospinal fluid
ERK1/2	Extracellular signal-regulated kinase 1, 2
FGF21	Fibroblast growth factor 21
GAS	Gamma-Activated Sequences
Glu	Glutamate
Gly	Glycine
GPR35	G protein–coupled receptor 35
GSK3	Glycogen synthase kinase 3
HIIT	High-Intensity Interval Training
HD	Huntington’s disease
HPA	Hypothalamic–pituitary–adrenal axis
IDO	indoleamine 2,3-dioxygenase
IFN-γ	Interferon-gamma
IL-6	Interleukin-6
IRF1	Interferon Regulatory Factor 1
JAK	Janus Kinase
KAT	Kynurenine aminotransferase
KMO	Kynurenine monooxygenase
KP	Kynurenine pathway
KYN	Kynurenine
KYNA	Kynurenic acid
KYNU	Kynureninase
LAT1	Large neutral amino acid transporters
MAO	Monoamine oxidase
MCT	Moderate continuous training
MDD	Major depressive disorder
mTOR	Mechanistic target of rapamycin
NAD^+^	Nicotinamide adenine dinucleotide
NAS	N-acetylserotonin
NFK	N-formylkynurenine
NF-κB	Nuclear factor kappa B
NMDAr	N-methyl-D-aspartate receptors
nNOS	Neuronal nitric oxide synthase
NO	Nitric oxide
NRF1	Nuclear respiratory factor 1
PD	Parkinson’s disease
PGC-1α	Peroxisome proliferator-activated receptor gamma coactivator-1α
PI3K	Phosphatidylinositol-3-kinase
PI3Kα	Phosphoinositide 3-kinase alpha
PPARα	Peroxisome proliferator-activated receptor alpha
PSD-95	Postsynaptic density protein 95
PXR	Pregnane X receptor
Pyr	Pyruvate
QA	Quinolinic acid
QAPRT	Quinolinic acid phosphoribosyltransferase
Raf	Rapidly Accelerated Fibrosarcoma kinase
Ras	Rat sarcoma oncogene family GTPase
ROS	Reactive oxygen species
SIRT1	Sirtuin 1
STAT1	Signal Transducer and Activator of Transcription 1
TDO	Tryptophan 2,3-dioxygenase
TFAM	Mitochondrial transcription factor A
TH2	T helper type 2 lymphocytes
TNF-α	Tumor necrosis factor-α
TPH	Tryptophan hydroxylase
TrkB	Tropomyosin receptor kinase B
Trp	Tryptophan
XA	Xanthurenic acid
α7 nAChR	α7 nicotinic acetylcholine receptor

## Appendix A

Stratify first: age/sex, body mass index (BMI)/Type 2 diabetes mellitus, estimated glomerular filtration rate (eGFR)/renal risk, and physical capacity.

Baseline biomarkers: KYN/Trp, QA/KYNA, CRP/IL-6/TNF-α, and Trp (±neurofilament light chain (NfL)).

Core prescription:

Aerobic 150–300 min/week (walk/jog/cycle)

HIIT 1–3×/week as tolerated

Resistance 2–3×/week

Mind–body (yoga/Tai Chi) to increase adherence/sleep

Progress loads are 5–10%/week if symptoms are stable.

Add phenotype-fit mimetics/adjuncts:

Atherometabolic dyslipidemia: Fibrate (PPARα); Type 2 diabetes mellitus (PPARγ) is considered if insulin resistance is present (watch edema/congestive heart failure (CHF)).

Type 2 diabetes mellitus/obesity or low capacity: Metformin (AMPK); consider NAD^+^ boosters/resveratrol (SIRT1) where appropriate.

High inflammatory/KP load: consider AdipoRon or KMO inhibitors only in protocol/clinical trial settings.

Cardiometabolic fatigue: Meldonium/trimetazidine was approved/appropriate.

Targets at 8–12 weeks:

KP: KYN/Trp ↓ ≥ 15–20%, QA/KYNA ↓ ≥ 20%, KYNA ↑

Inflammation: CRP ↓ ≥ 30% (hs-CRP < 2 mg/L if possible)

Function: VO_2_ peak ↑ ≥ 10% or 6-min walk test (6MWT) +40 m

Symptoms: Patient Health Questionnaire–9 items (PHQ-9) −5 points (or ≥50% ↓), fatigue ↓, Montreal Cognitive Assessment (MoCA) +2 if low at baseline.

Adaptive:

Met targets: maintain and de-escalate adjuncts.

Partial: add one HIIT/resistance exercise training session/week or introduce a phenotype-fit mimetic.

Nonresponse/intolerance: switch modality; supervised rehab; consider clinical trials.

Safety essentials: eGFR before/during metformin; B12 yearly if on metformin; thiazolidinediones cautions (edema/CHF, fractures); drug–drug checks; hydration/nutrition; periodized loads; warm-up/cool-down.
